# 697 Novel Approach to 3D Printing for Early Pre-prosthetic Adaptation for a Burn Survivor

**DOI:** 10.1093/jbcr/iraf019.326

**Published:** 2025-04-01

**Authors:** David Chung, Adrianna Olch, Theresa Chin, Jennifer Bailey, Jones Seto, Syed Saquib, Natalya Godes

**Affiliations:** University of California Irvine Medical Center; University of California Irvine Medical Center; University of California Irvine Medical Center; University of California Irvine Medical Center; University of California Irvine Medical Center; University of California Irvine Medical Center; University of California Irvine Medical Center

## Abstract

**Introduction:**

Deep burns or traumatic injuries requiring upper extremity (UE) amputation can severely limit a patient’s ability to participate with functional activities in acute and early rehabilitation phases of recovery. This case study describes the impact of initiating a preparatory UE prosthesis early in the acute care phase of recovery for a patient with an UE amputation. It demonstrates the significant impact of 3D printed attachments as a low cost and accessible option used with a prosthesis to maximize patient participation in self-care activities and accelerate adaptation as a new amputee.

**Methods:**

The patient is a 46-year-old male who sustained 51% TBSA flame burn, requiring a transradial UE amputation and his opposing arm was 3rd and 4th degree burns. Patient movement patterns and range of motion (ROM) were analyzed to determine the most versatile and efficient attachments to be used with the prosthesis. The 3D printed hook was based on an open-source design and modified to fit the prosthesis. Progress was determined by weekly ROM measurements and the Inpatient Boston AM-PAC “6-click” for Activities of Daily Living (ADL). Subjective patient statements and qualitative reports of patient mood and participation were also collected from psychologist and therapist notes.

**Results:**

Prior to receiving the prosthesis, the patient was dependent for ADLs, expressed sadness regarding functional status, with low motivation to participate with therapies. After initiating the device, he improved to minimal assist (minA) for self-feeding but remained total assist for other ADLs. A body powered cable system and 3D hook was fabricated and attached to the prosthesis and he improved to a setup level for self-feeding, minA for hygiene and grooming, and moderate assistance for lower body dressing. Patient’s shoulder flexion also improved from 90 to 140 degrees. After receiving the attachments for the prosthesis, he expressed feelings of accomplishment and motivation to continue rehabilitation due to his improved independence for self-care. The overall cost of the 3D printed materials was $20. Significant improvement with ADLs was demonstrated with a Boston AM-PAC score of 6 at initial evaluation and 13 at discharge.

**Conclusions:**

Demonstrated with this case study, 3D printing prosthetic attachments is a low cost solution that provides better access to prosthetic accessories, accelerating the progress of a new amputee to perform ADLs. This innovative use of 3D printing allowed the patient to better qualify for inpatient rehabilitation after discharge and improve the psychosocial state of the patient.

**Applicability of Research to Practice:**

3D printing in conjunction with a prefabricated prosthetic can allow for increased early participation in ADLs for patients experiencing UE amputation and large TBSA burns. 3D printing has the potential to offer low cost and more easily accessible alternatives to expensive prosthetics accelerating rehabilitation physically and psychosocially.

**Funding for the Study:**

N/A

